# Supplementary material for: Human Serum and Salivary Metabolomes: Diversity and Closeness
Source: Int J Mol Sci. 2023 Nov 22;24(23):16603. doi: 10.3390/ijms242316603 (PMC10706786; doi:10.3390/ijms242316603)
Supplement: Supplementary file 1 [file ijms-24-16603-s001.zip › ijms-2714984-supplementary.pdf]

Article

The human serum and salivary metabolomes: diversity and closeness

Elena Ferrari[1], Mariana Gallo[1*], Alberto Spisni[1], Rita Antonelli[2], Marco Meleti[2], Thelma A. Pertinhez[1]

[1] Laboratory of Biochemistry and Metabolomics, Department of Medicine and Surgery, University of Parma, Parma, Italy
[2] Centro Universitario Odontoiatria, University of Parma, Parma, Italy
* Correspondence: Mariana Gallo, E-mail: mariana.gallo@unipr.it

Table S1. Metabolites profiles of the three salivary types and serum (mean concentrations ± SD).

Table S2. Demographic data and salivary sample collection rates of the enrolled subjects.

Figure S1. Comparison of the mean concentrations of the metabolites shared by PS and SM/SLS samples.

Figure S2. Mean fold changes ≥ 1 for metabolites shared by serum and saliva samples.

Figure S3. Amino acid fractional abundance of serum, PS, SM/SLS, and WS samples.

Table S1. Metabolites profiles of the three salivary types and serum (mean concentrations ± SD)

Metabolite	WS (µM)	PS (µM)	SM/SLS (µM)	Serum (µM)
**Organic acids**				
2-Aminoadipate	**226.5 ± 130.8**[a]	38.7 ± 35.7	12.3 ± 7.1	-[b]
2-Hydroxy-3-methylvalerate	10.9 ± 7.3	-	-	-
2-Hydroxybutyrate[c]	15.7 ± 10.9	7.9 ± 2.5	2.6 ± 1.3	**42.6 ± 16.9**
2-Hydroxyisovalerate	2.3 ± 0.9	2.6 ± 1.2	-	**8.1 ± 3.9**
3-Hydroxyisobutyrate	-	2.4 ± 0.8	-	-
3-Methylglutarate	18.9 ± 11.1	-	-	-
3-Phenylpropionate	13.2 ± 10.2	-	-	-
4-Hydroxyphenylacetate	8.8 ± 5.6	-	-	-
4-Hydroxyphenyllactate	7.9 ± 6.3	-	-	-
5-Aminopentanoate	**168.8 ± 104.2**	26.2 ± 25.8	9.1 ± 11.7	-
Acetate	**2667.2 ± 1123.6**	676.4 ± 730.6	382.0 ± 329.4	50.0 ± 17.0
Butyrate	**30.1 ± 23.6**	6.1 ± 4.1	3.8 ± 2.1	-
Caprylate	-	-	5.5 ± 3.0	-
Citrate	14.9 ± 8.9	43.7 ± 25.4	28.8 ± 29.6	**108.2 ± 17.8**
Formate	**60.3 ± 54.7**	27.2 ± 26.8	24.3 ± 19.6	12.4 ± 3.9
Fumarate	-	2.9 ± 1.0	-	-
Glycolate	-	-	12.2 ± 12.3	-
Isobutyrate	21.2 ± 11.5	-	-	-
Isocaproate	9.2 ± 6.0	-	-	-
Isovalerate	9.5 ± 6.6	-	-	-
Lactate	156.3 ± 121.5	798.7 ± 335.6	172.8 ± 99.1	**1763.1 ± 470.3**
Malonate	**7.6 ± 5.1**	6.8 ± 5.6	-	-
Phenylacetate	**18.0 ± 12.0**	-	2.1 ± 0.7	-
Propionate	**313.8 ± 167.7**	48.6 ± 49.6	19.5 ± 15.0	-
Pyruvate	21.7 ± 12.7	35.7 ± 55.6	5.9 ± 3.2	**56.1 ± 27.2**
Succinate	**17.8 ± 8.2**	13.2 ± 6.1	5.6 ± 2.3	-
**Ketone bodies**				
3-Hydroxybutyrate	6.6 ± 3.4	8.2 ± 4.4	4.3 ± 2.2	**97.6 ± 69.8**
Acetoacetate	-	-	-	43.9 ± 24.7
Acetone	-	-	-	21.5 ± 9.6
**Amino acids and derivatives**				
Alanine	44.2 ± 46.9	81.6 ± 40.2	24.8 ± 15.4	**264.8 ± 63.8**
Alloisoleucine	**6.2 ± 3.3**	3.4 ± 1.4	-	-
Arginine	-	27.4 ± 14.9	-	**43.5 ± 9.4**
Asparagine	-	-	-	22.8 ± 6.1

Aspartate	29.3 ± 18.4	**29.4 ± 18.6**	7.6 ± 3.8	14.4 ± 2.7
Betaine	2.1 ± 0.7	7.6 ± 5.4	2.4 ± 1.8	**31.7 ± 11.0**
Carnitine	**5.6 ± 3.5**	-	4.1 ± 2.1	-
Creatine	15.7 ± 12.6	**53.1 ± 21.5**	19.1 ± 9.1	22.3 ± 12.7
Creatinine	5.7 ± 2.7	5.9 ± 2.8	4.0 ± 1.8	**68.6 ± 12.6**
Glutamate	**109.9 ± 63.0**	79.8 ± 54.9	23.8 ± 15.4	15.5 ± 8.1
Glutamine	46.6 ± 35.7	72.5 ± 36.3	26.5 ± 14.7	**448.0 ± 77.5**
Glycine	133.3 ± 104.0	83.1 ± 64.4	22.4 ± 17.5	**180.2 ± 38.8**
Histidine	24.3 ± 13.8	23.2 ± 17.4	7.6 ± 4.8	**75.9 ± 17.6**
Homoserine	-	**47.6 ± 32.3**	32.7 ± 18.2	-
Isoleucine	7.8 ± 8.8	5.5 ± 3.5	-	**50.2 ± 7.9**
Leucine	18.5 ± 15.9	14.5 ± 7.0	5.5 ± 2.1	**97.4 ± 17.7**
Lysine	73.5 ± 44.2	31.4 ± 23.4	6.2 ± 3.5	**119.2 ± 22.8**
Methionine	-	-	2.5 ± 0.8	**16.9 ± 2.7**
O-Acetylcarnitine	-	-	-	6.6 ± 2.1
Ornithine	-	-	-	36.7 ± 13.0
Phenylalanine	23.5 ± 13.1	14.3 ± 9.5	5.4 ± 2.7	**42.1 ± 5.9**
Proline	138.3 ± 136.6	55.1 ± 44.1	7.5 ± 4.6	**189.3 ± 72.6**
Putrescine	**45.7 ± 29.7**	8.2 ± 7.3	-	-
Pyroglutamate	16.8 ± 16.5	-	7.6 ± 3.1	**28.6 ± 7.9**
Sarcosine	**4.6 ± 2.5**	4.5 ± 2.8	-	-
Serine	-	-	-	98.5 ± 21.1
Taurine	50.9 ± 30.3	**169.6 ± 90.5**	65.3 ± 39.4	91.1 ± 32.4
Threonine	8.1 ± 7.8	-	8.5 ± 4.4	**88.4 ± 17.7**
Tyrosine	43.6 ± 24.6	34.0 ± 19.8	12.1 ± 6.1	**50.1 ± 9.6**
Urea	120.9 ± 206.4	**581.3 ± 384.4**	351.7 ± 287.6	265.8 ± 100.0
Valine	16.2 ± 16.0	17.3 ± 10.8	4.7 ± 2.7	**187.5 ± 34.5**
β-Alanine	-	**4.2 ± 2.1**	1.9 ± 0.8	-
**Carbohydrates and derivatives**				
Fucose	**61.2 ± 73.4**	16.0 ± 18.3	6.6 ± 4.4	-
Galactose	27.3 ± 23.3	-	-	-
Glucose	23.2 ± 28.9	256.7 ± 180.0	63.1 ± 47.1	**3751.0 ± 265.4**
Lactose	**30.5 ± 24.1**	-	26.3 ± 13.6	-
Maltose	7.8 ± 12.9	**404.5 ± 336.3**	95.4 ± 85.6	-
Mannose	-	-	-	37.0 ± 5.6
N-Acetylglucosamine	**52.9 ± 42.5**	-	14.2 ± 12.7	-
myo-Inositol	-	-	-	14.5 ± 4.7
**Nitrogenous bases and derivatives**				

1,7-Dimethylxanthine	-	4.6 ±4.7	-	-
3-Methylxanthine	-	5.0 ± 3.1	2.9 ± 1.9	-
AMP	-	**9.4 ± 5.6**	4.0 ± 2.6	-
Hypoxanthine	4.9 ± 4.5	**16.8 ± 12.0**	5.1 ± 2.4	-
Uracil	6.3 ± 5.6	**6.7 ± 5.3**	-	
Uridine	-	4.1 ± 2.3	-	
Xanthine	6.3 ± 5.0	**11.7 ± 11.3**	7.6 ± 7.6	-
Xanthosine	-	2.8 ± 1.1	-	-
**Others**				
Choline	7.5 ± 4.6	**9.7 ± 5.8**	3.4 ± 1.5	7.2 ± 1.2
Ethanolamine	28.4 ± 25.9	**45.5 ± 48.9**	8.7 ± 7.9	-
Glycerol	107.1 ± 30.7	102.3 ± 29.3	53.5 ± 10.8	**161.3 ± 38.8**
Methanol	-	-	-	37.6 ± 16.6
O-Phosphocholine	-	10.4 ± 4.0	-	-
O-Phosphoethanolamine	34.5 ± 18.9	**130.2 ± 49.6**	59.2 ± 24.4	
Trimethylamine	-	8.6 ± 4.6	-	-
sn-Glycero-3-phosphocholine	-	-	-	33.9 ± 9.4

[a] When a metabolite is present at least in two sample matrices, the highest mean concentration is in bold; [b] not quantified; [c] when a metabolite is present in all sample matrices, its name is underlined.

Table S2. Demographic data and salivary sample collection rates of the enrolled subjects

Subjects	Age	Sex	*sample collection rate (mL/min)*		
			**PS**	**SM/SLS**	**WS**
01	25	*M	0.05	0.12	0.39
02	25	*F	0.08	0.23	0.27
03	24	M	0.36	0.39	0.68
04	23	M	0.08	0.32	0.68
05	25	F	0.10	0.18	0.68
06	25	M	0.11	0.20	0.15
07	25	F	0.11	0.15	0.90
08	24	F	0.07	0.15	0.54
09	25	M	0.27	0.23	0.54
10	25	F	0.05	0.15	0.25
11	21	M	0.18	0.14	0.25
12	24	F	0.12	0.26	1.08
13	25	F	0.12	0.28	0.39
14	23	M	0.19	0.34	1.08
15	23	M	0.06	0.08	0.23
16	23	M	0.05	0.11	0.30
17	20	F	0.06	0.10	0.39
18	24	F	0.05	0.12	0.26
19	20	F	0.07	0.16	0.39
20	25	M	0.12	0.36	0.49
**mean±SD**	**23.7±1.6**		**0.11±0.08**	**0.20± 0,09**	**0.49±0.28**

* M/F, male/female. The male and female distributions of saliva collection rates of PS, SM/SLS and WS are not significantly different at the 0.05 level (Mann Whitney test).

(A)

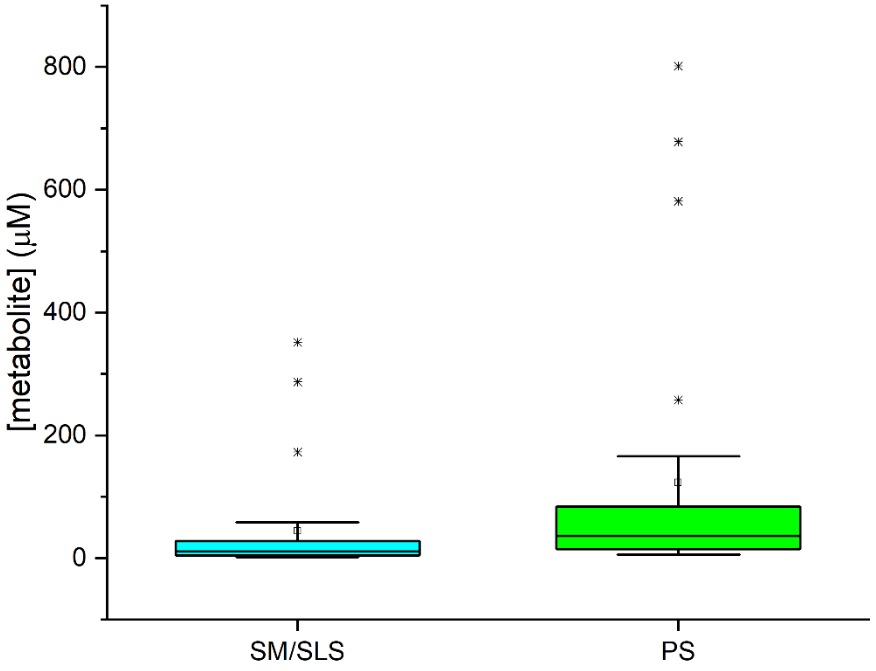

(B)

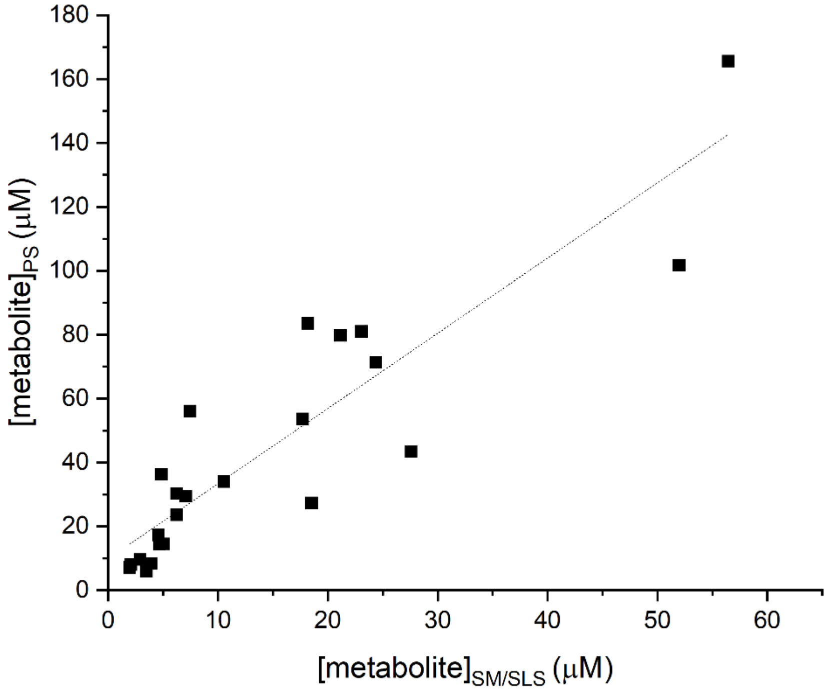

Figure S1. Comparison of the mean concentrations of the metabolites shared by PS and SM/SLS samples. (A) Distributions of the metabolite mean concentrations. The boxes are determined by the 25th and 75th percentiles; the line inside the boxes and the square are the median and the mean values, respectively; the asterisks identify the outliers (Acetate, Lactate, Urea for SM/SLS and PS; Glucose only for PS). (B) Comparison of the mean metabolite concentrations after eliminating the outliers. The dashed line is the regression line: $y=9.8+2.3x$; $R^2=0.81$.

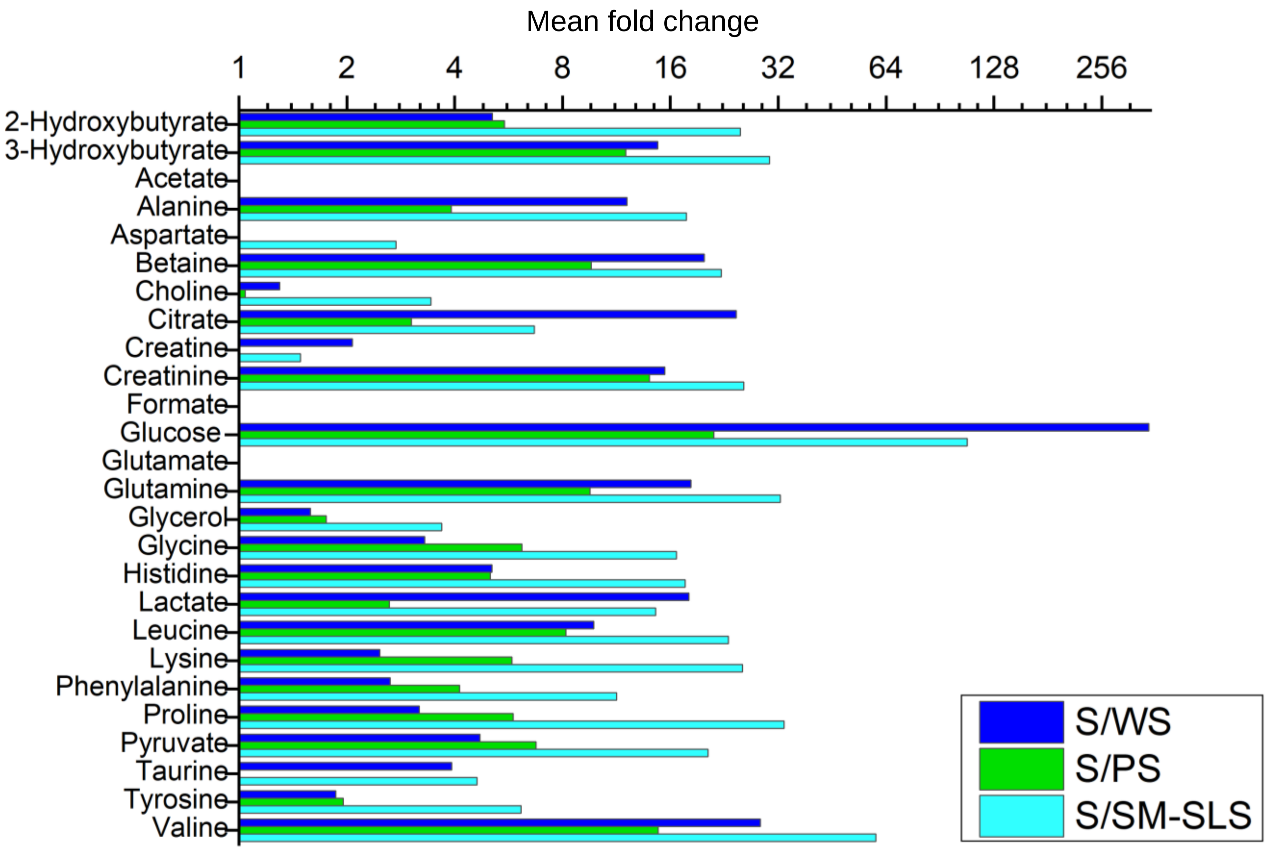

Figure S2. Mean fold changes ≥ 1 for metabolites shared by serum and saliva samples. Mean fold changes were obtained by averaging the ratios obtained by dividing the metabolite concentration in serum by its concentration in PS, SM/SLS or WS of the 20 study subjects. Missing bars correspond to mean fold changes lower than 1. On Y axis are the metabolites shared by WS, PS, SM/SLS, and serum. X axis is in log_2 scale.

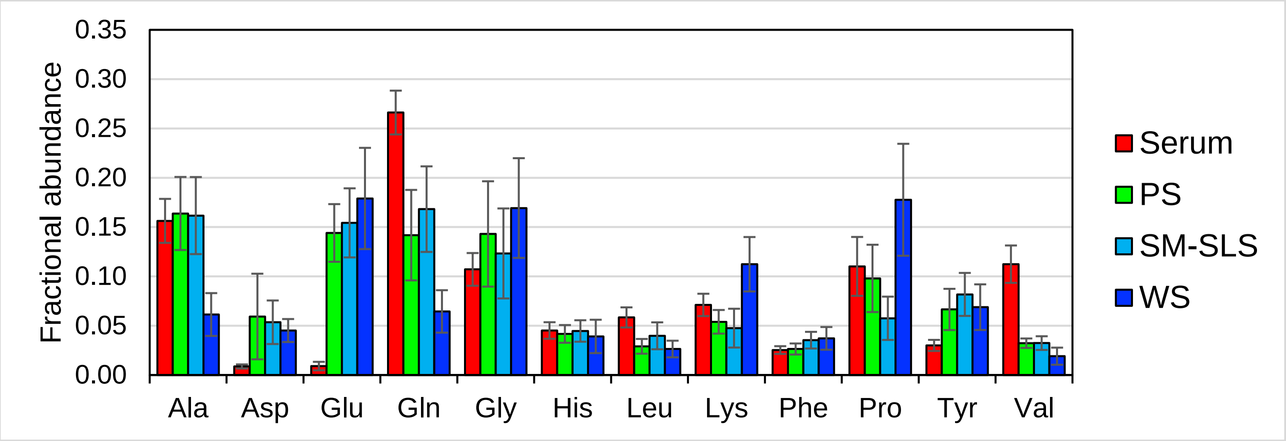

Figure S3. Amino acid fractional abundance of serum, PS, SM/SLS, and WS samples. The concentration of the amino acid of interest has been divided by the sum of the concentrations of all amino acids present in the sample matrix. The bar plot shows the average values ± SD.
